# mem-iLID, a fast and economic protein purification method

**DOI:** 10.1042/BSR20210800

**Published:** 2021-06-28

**Authors:** Ruijing Tang, Shang Yang, Georg Nagel, Shiqiang Gao

**Affiliations:** Department of Neurophysiology, Institute of Physiology, Biocenter, University of Wuerzburg, Wuerzburg 97070, Germany

**Keywords:** light-induced dimerization, membrane anchor, Optogenetics, protein purification

## Abstract

Protein purification is the vital basis to study the function, structure and interaction of proteins. Widely used methods are affinity chromatography-based purifications, which require different chromatography columns and harsh conditions, such as acidic pH and/or adding imidazole or high salt concentration, to elute and collect the purified proteins. Here we established an easy and fast purification method for soluble proteins under mild conditions, based on the light-induced protein dimerization system improved light-induced dimer (iLID), which regulates protein binding and release with light. We utilize the biological membrane, which can be easily separated by centrifugation, as the port to anchor the target proteins. In *Xenopus laevis* oocyte and *Escherichia coli*, the blue light-sensitive part of iLID, *As*LOV2-SsrA, was targeted to the plasma membrane by different membrane anchors. The other part of iLID, SspB, was fused with the protein of interest (POI) and expressed in the cytosol. The SspB-POI can be captured to the membrane fraction through light-induced binding to *As*LOV2-SsrA and then released purely to fresh buffer in the dark after simple centrifugation and washing. This method, named mem-iLID, is very flexible in scale and economic. We demonstrate the quickly obtained yield of two pure and fully functional enzymes: a DNA polymerase and a light-activated adenylyl cyclase. Furthermore, we also designed a new SspB mutant for better dissociation and less interference with the POI, which could potentially facilitate other optogenetic manipulations of protein–protein interaction.

## Introduction

The purification of recombinant proteins has increased enormously in recent years with the intensive demands of protein characterization and engineering. Popular purification techniques are based on precipitation with pH, salts, or temperature, ion exchange, hydrophobic chromatography or affinity chromatography. Because of the highly specific biorecognition, affinity chromatography is now widely used. Affinity tags, either small peptides like FLAG, poly-His and c-myc or larger protein domains like CBP, SBP, GST and protein G, are genetically fused to the target proteins and lead to specific binding to the desired ligand [1]. The bound tag–ligand pairs can be then detached by changing ion concentration or adding other competitive molecules to release the purified protein.

Over the years, column-free protein purification has been proposed and utilized, based on aggregating tags. N^pro^ is a highly hydrophobic protein with 168 residues [2], which has been used as an aggregating tag to increase the expression level of target protein in the form of inclusion body. Some short self-assembling peptide tags such as ELK16, L_6_KD and GFIL8 [3] and protein tags like CipA [4] can also induce protein aggregates. Unlike conventional inclusion bodies, these proteins are highly active. ELP and BRT17 [5], tag-based strategies have also been used to co-express target proteins in the soluble fraction which aggregate *in vitro*, mediated by (NH_4_)_2_SO_4_ and CaCl_2_ for protein separation. Non-chromatographic purification based on aggregating tags reduces the purification processes and the experimental costs at laboratory scale. However, the self-cleaving inteins such as △I-CM, DnaE and DnaB should be co-expressed with aggregating tags for self-cleaving, induced by pH shift and/or temperature changes to remove the aggregating tags and restore the target proteins to the soluble phase [4,5].

Light-gated protein dimerization systems have been developed and applied in the optogenetics field [6–8]. Optogenetics is a rapid, reversible and non-invasive technique that controls protein function and thus cell physiology with light, developed and widely used after the discovery of channelrhodopsin [9,10]. Light-induced protein dimerization systems were developed from natural photoreceptors like cryptochrome [11], LOV (light, oxygen, or voltage) domain-containing photoreceptors [12,13], and phytochrome [14] etc.

The Cry2/CIB1 pair is developed from plant photoreceptor cryptochrome 2 (Cry2) which shows blue light-induced dimerization with CIB1. The Cry2/CIB1 system has been used for optogenetic control of protein activity and translocation [15–17]. It is noted that Cry2 oligomerizes into large clusters under blue light in addition to associating with CIB1 [18]. This character has been applied to optogenetic control of phase transitions [19] but could be a drawback for applications that require precise stoichiometry. Another dimerization pair—PhyB/PIF—is also developed from a natural plant photoreceptor [20]. The red-light photoreceptor phytochrome B (PhyB) could be activated by red light to interact with PIF and dissociate when exposed to far-red light.

The improved light-induced dimer (iLID) [13] utilizes the LOV2 domain of phototropin 1 from *Avena sativa* (*As*) as the photoactive element and incorporates a naturally binding pair from *Escherichia coli*: seven amino acids of SsrA peptide and its binding partner SspB, a 13-kDa adaptor protein [21]. In the dark, the SsrA peptide, fused to AsLOV2, is sterically blocked from binding SspB. When illuminated with blue light, the C-terminal Jα helix of the LOV2 domain undocks from the protein, allowing the SsrA peptide to bind SspB. The iLID system was used for optogenetic manipulation of different cells by light-driven protein dimerization or protein localization to specific subcellular domains [22,23]. Due to the high binding affinity of the heterodimer pair SsrA/SspB, the iLID system (with the original SspB, SspB_nano) showed undesirable dark activity, i.e. binding in the dark [22,24]. Thus, other optimized iLIDs, such as iLID_micro and iLID_milli, were designed by point mutations in SspB to decrease the binding affinity and to meet the requirements of different experimental purposes [25].

In *Xenopus laevis* oocytes, the blue light-sensitive part of iLID, *As*LOV2-SsrA (LOV-A for short in the present study), was targeted to the plasma membrane by the short membrane anchor Lyn11 [26]. The other part of iLID, SspB, was fused with the protein of interest (POI) and expressed in the cytosol. The SspB-POI can be captured to the membrane fraction through light-gated binding to LOV-A and then released purely to a fresh buffer in the dark after simple centrifugation and washing.

In *E. coli*, the plasma membrane anchor was changed to helix1021 (H1021 for short in this manuscript) [27]. Furthermore, we also developed a new monomeric SspB mutant showing better dissociation rate and less interference with the POI function. After characterization of the light-assisted purification method with YFP, we further applied this method to purify the bacteria photo-activated adenylyl cyclase (bPAC) [28] and a DNA polymerase, proving a fast and flexible protein purification process.

The mem-iLID method we developed is easy, economic and very flexible in scale. It will facilitate extensive protein engineering and purification of proteins sensitive to harsh buffer conditions. Our newly engineered iLID pair could also facilitate other optogenetic applications of light-gated protein interactions.

## Results

### Light-assisted protein purification based on iLID in *Xenopus* oocytes

**Figure 1:**
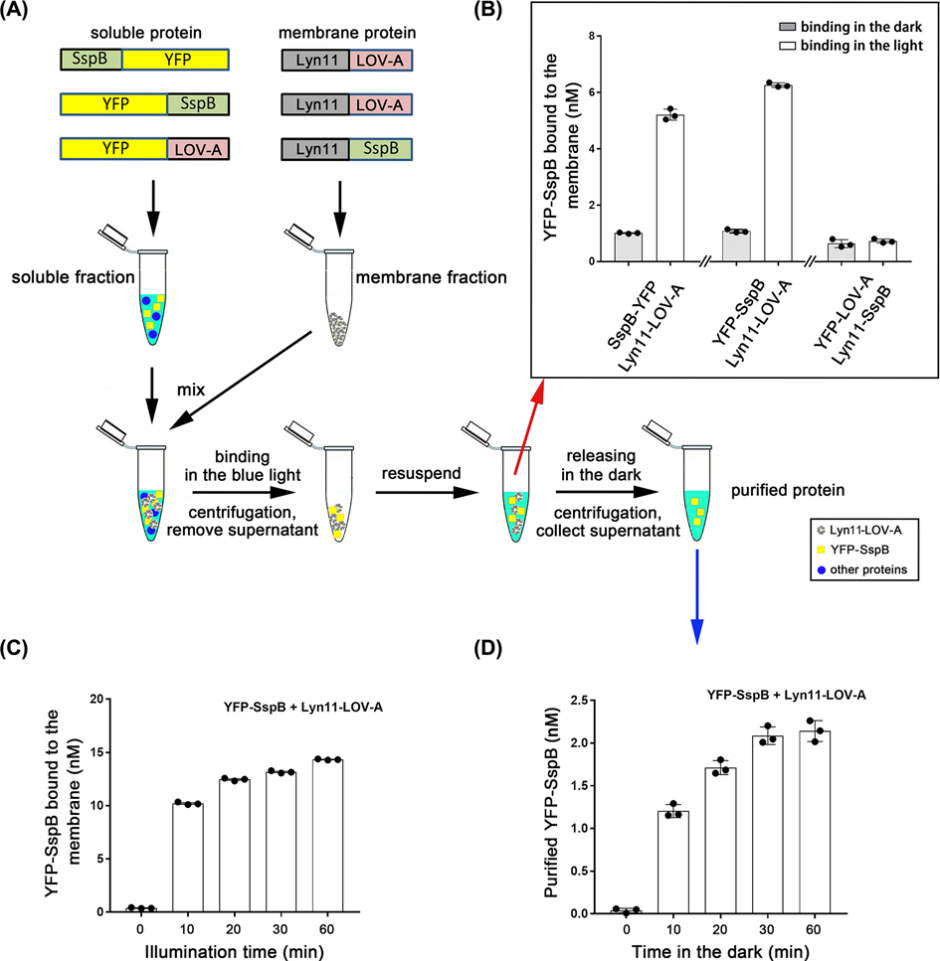
Light-assisted protein purification based on iLID/SspB after *Xenopus* oocyte expression (**A**) SspB (the original SspB, which is also called SspB_nano) and iLID were fused with YFP and expressed as soluble fraction, or fused with Lyn11 and expressed as membrane fraction, separately. The general purification process was shown in the schematic diagram. The detailed protocol was described in the ‘Materials and methods’ part. (**B**) The amount of YFP-tagged proteins bound to the membrane fractions after 30 min in the dark or blue light illumination. The red arrow indicated the corresponding step of the tested samples. For each reaction, membrane fraction from 25 oocytes was mixed with soluble fraction containing a final 20 nM YFP-tagged proteins in 300 μl buffer A. *n*=3, error bars = SEM, all individual data points are shown. (**C**) Illumination time-dependent binding efficiency of Lyn11-LOV-A and YFP-SspB. Illumination was performed with blue light. Each reaction contained a soluble fraction of 40 nM YFP-SspB and membrane fraction from 25 oocytes in 300 μl buffer A. *n*=3, error bars = SEM, all individual data points are shown. (**D**) Time-dependent release efficiency of YFP-SspB from the membrane expressing Lyn11-LOV-A in the dark. The binding was induced by 20 min blue light of (C). The blue arrow indicated the corresponding step of the tested samples. *n*=3, error bars = SEM, all individual data points are shown.

We developed an *in vitro* system to test the binding efficiency of LOV-A (abbreviation for *As*LOV2-SsrA in the present study) and SspB, both expressed in *Xenopus* oocytes. We started using the SspB_nano version that has a high binding affinity with LOV-A (4.7 μM in the dark and 0.132 μM in the light) [13]. The LOV-A part was fused with the membrane anchor Lyn11 (Lyn11-LOV-A) for membrane-targeted expression in the oocyte. The SspB_nano part was fused with YFP and expressed in the cytosol (Figure 1A). After 2 days of expression in the dark, 5 min low-speed (500×***g***) centrifugation was applied to remove nuclei, mitochondria, and cell debris of homogenized oocytes. The membrane fraction and soluble fraction were then separated by 1.5 min high-speed (15000×***g***) centrifugation. The isolation process was performed in red light to keep LOV-A in the caged stage. The soluble fraction containing YFP-SspB_nano or SspB_nano-YFP was mixed with the membrane fractions expressing Lyn11-LOV-A and placed in the dark or under the blue light (470 nm, 100 μW/mm^2^) illumination (Figure 1A). After 30-min illumination, the re-collected membrane fraction exhibited approximately six-fold YFP fluorescence increase, compared with that in the dark (Figure 1B), indicating the binding of LOV-A and SspB_nano in the light. We then exchanged the position of LOV-A and SspB_nano: SspB_nano was anchored to the oocyte membrane by Lyn11 and LOV-A was fused with YFP as a soluble protein, but this combination exhibited weak binding efficacy (Figure 1B). Therefore, YFP-SspB_nano and Lyn11-LOV-A were chosen for further study within the *Xenopus* oocyte system.

We next checked the time-dependent binding efficacy between YFP-SspB_nano and Lyn11-LOV-A. Ten-minute min illumination already induced the binding dramatically. Twenty-minute illumination increased the binding further while longer times improved the binding efficacy moderately (Figure 1C). Whereas long-time blue light exposure might lead to irreversible photochemical reactions of the LOV2 domain in the dark [29], we finally chose 20-min illumination time considering both the binding efficacy and potential photo damage.

The time-dependent release of YFP-SspB_nano from Lyn11-LOV-A was then tested in the dark. The membrane fraction containing Lyn11-LOV-A and the bound YFP-SspB_nano was washed and resuspended in fresh buffer, and then placed in the dark for different times (Figure 1A). After 1.5 min high-speed (15000×***g***) centrifugation, the released YFP-sspB_nano in the soluble fraction was collected and monitored by the YFP fluorescence. Thirty minutes in the dark reached the maximum release, and 10 min in the dark released more than half of that (Figure 1D).

Our experiments showed successful light-controlled binding and release with iLID, when expressed in *Xenopus* oocytes. As oocytes are a fast and convenient test system but not useful for large-scale production, we next tested expression in *E. coli*.

### Light-assisted protein purification after expression in *E. coli*

**Figure 2:**
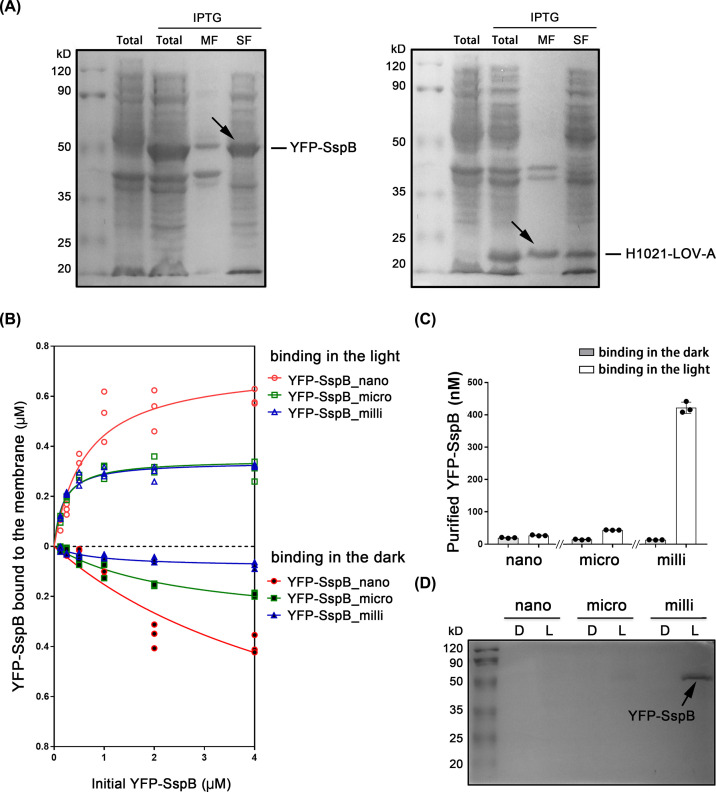
Light-assisted protein purification after *E. coli* expression (**A**) Expressions of YFP-SspB (original SspB, SspB_nano) and H1021-LOV-A were induced by 0.5 mM IPTG and confirmed in SDS/PAGE. A total of 50 ml *E. coli* culture was washed and homogenized in 1 ml buffer A for the crude extraction of membrane fraction and soluble fraction. The extracted membrane fractions were resuspended in 1 ml buffer A. Five microliters of each sample was loaded to the SDS/PAGE. Total: the whole cell lysate after ultrasonic homogenization. MF, membrane fraction. SF, soluble fraction. The YFP-SspB (45 kDa) band in soluble fraction and H1021-LOV-A (24 kDa) band in membrane fraction were indicated by black arrows. (**B**) The amounts of different YFP-SspB versions bound the membrane fractions containing H1021-LOV-A were compared after 20 min binding in the dark or blue light illumination. In each reaction, the membrane fraction from 2.5 ml *E. coli* culture was mixed with different concentrations of YFP-SspB in 300 μl buffer A. Michaelis–Menten curves were fitted. *n*=3, all individual data points are shown. (**C**) The amounts of purified proteins with different YFP-SspB versions were compared. The membrane fraction from 10 ml *E. coli* was mixed with soluble fraction containing 4 μM YFP-SspB for the purification. The purified proteins were collected in 200 μl buffer A. Illumination time is 20 min and releasing time in the dark is 30 min. *n*=3, error bars = SEM, all individual data points are shown. (**D**) Ten microliters purified YFP-SspB from (**C**) were tested in the SDS/PAGE. The YFP-SspB band was indicated by the black arrow. D, binding in the dark. L, binding in the light.

We changed the membrane anchor of LOV-A to the transmembrane helix1021 (H1021), a 38-amino acid portion of the open reading frame sll1021 from the cyanobacterium *Synechocystis* sp. PCC6803, previously tested in *E. coli* [27]. The YFP-SspB_nano and H1021-LOV-A were separately inserted into the pET28b vector and transformed into *E. coli* BL21(DE3). The soluble fraction and membrane fractions of *E. coli* were separated by centrifugation after ultrasonic homogenization. For YFP-SspB_nano-expressing *E. coli*, an expected band of 45 kDa from supernatant was seen in the soluble fraction after 0.5 mM isopropyl-β-d-thiogalactopyranoside (IPTG) induction (Figure 2A), whereas H1021-LOV-A protein was detected in both the membrane and soluble fraction of corresponding cells. The size of 24 kDa confirmed the partial membrane targeting of H1021-LOV-A in *E. coli* (Figure 2A).

However, we found that the bound YFP-SspB_nano is not released efficiently in the dark, due to the high dark affinity and the increased protein content (100-times more than in the *Xenopus* oocyte system) (Figures 1B and 2B,C). For SspB, two other variants were published: SspB_micro (binding affinity 47 μM in the dark and 0.8 μM in the light) and SspB_milli (binding affinity > 1 mM in the dark and 56 μM in the light) [25]. We then tested the SspB_micro and SspB_milli after expression in *E. coli* BL21(DE3). The binding ability of YFP-SspB_nano/H1021-LOV-A, YFP-SspB_micro/H1021-LOV-A and YFP-SspB_milli/H1021-LOV-A in the light and dark were compared (Figure 2B). Different amounts of YFP-SspB were mixed with the extracted H1021-LOV-A-expressing *E. coli* membrane. As shown in Figure 2B, SspB_nano exhibited very high binding ability in the dark with an L/D ratio of ∼1.5 at 4 μM YFP-SspB_nano concentration. The binding ability of SspB_micro and SspB_milli with H1021-LOV-A was similar in the light, whereas SspB_milli showed lower binding ability in the dark. At 4 μM YFP-SspB level, the L/D ratio for SspB_micro and SspB_mili was ∼1.5 and 6, respectively.

Soluble fraction containing 4 μM YFP-SspB and membrane fraction containing H1021-LOV-A from 10 ml *E. coli* culture were then mixed and placed in the dark or blue light illumination for 20 min. The membrane fraction was then collected by centrifugation, washed three times, resuspended in 200 μl fresh buffer and moved into dark to release the bound YFP-SspB. As shown in Figure 2C, the YFP-SspB_milli exhibited the most robust release ability in the dark. Then, 10 μl of each purified sample was loaded in the SDS/PAGE to check the purity. As shown in Figure 2D, only one band of YFP-SspB_milli with the right size can be seen in the light group, indicating that H1021-LOV-A/SspB_milli can be used for optogenetic protein purification with the *E. coli* system.

### Engineering and characterization of a monomeric SspB variant

**Figure 3:**
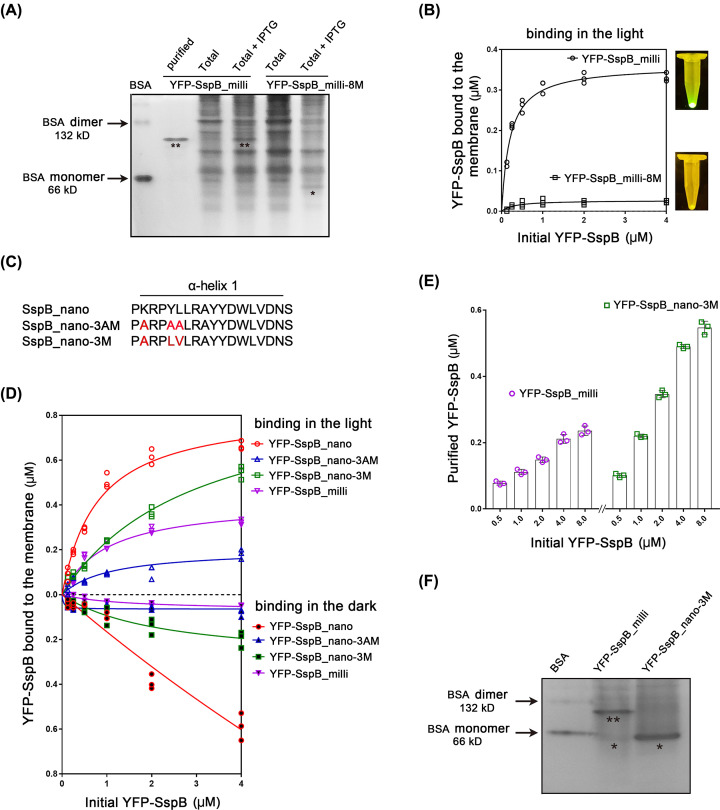
Generation and comparison of new monomeric SspB mutants (**A**) The extracted and *E. coli* whole cell lysate expressing YFP-SspB_milli and YFP-SspB_milli-8M in a native PAGE gel. The whole cell lysates were prepared from 50 ml *E. coli* culture resuspended and homogenized in 1 ml buffer A. Five microliters from each sample was loaded to the native PAGE. The gel was stained by EZBlue. Total: the whole cell lysate after ultrasonic homogenization. BSA was loaded as a marker, which is 66 kDa in monomeric state and 132 kDa in dimeric state (*: monomer, **: dimer). (**B**) The binding efficacy of YFP-SspB_milli (dimeric) and YFP-SspB_milli-8M (monomeric) to H1021-LOV-A after 20 min blue illumination. In each reaction, the membrane fraction from 2.5 ml *E. coli* was mixed with soluble fractions with different concentrations of YFP-SspB (different versions) in 300 μl buffer A. Michaelis–Menten curves were fitted. *n*=3, all individual data points are shown. (**C**) Sequences of the mutation part (α-helix1) for the newly designed SspB_nano mutations. The changed amino acids were labeled in red. (**D**) Comparing the binding abilities of different YFP-SspB mutants to H1021-LOV-A after 20 min in the dark or blue light illumination. In each reaction, the membrane fraction from 2.5 ml *E. coli* was mixed with different concentrations of YFP-SspB mutants in 300 μl buffer A. Michaelis–Menten curves were fitted. *n*=3, all individual data points are shown. (**E**) Comparing the purification efficacy of YFP-SspB_milli and YFP-SspB_nano-3M. The membrane fraction extracted from 5 ml *E. coli* was mixed with soluble fractions containing different initial concentrations of YFP-SspB in 300 μl buffer A for the purification. Illumination time is 20 min and releasing time in the dark is 30 min. The final purified proteins were collected in 200 μl buffer A. *n*=3, error bars = SEM. All individual data points are shown. (**F**) The purified YFP-SspB_milli and YFP-SspB_nano-3M from (E) were checked in a native PAGE gel. A total of 200 ng of each sample was loaded (*: monomer, **: dimer), BSA was loaded as a marker.

Although the light-induced dimer set H1021-LOV-A/SspB_milli was used for optogenetic protein purification successfully, it should be pointed out that SspB naturally forms a strong homodimer, which might influence the activity of some target proteins (see later part about bPAC activities after fusing with different SspB variants). To check the oligomeric state of YFP-SspB_milli, the purified YFP-SspB_milli was loaded in a native PAGE gel together with the lysate of *E. coli* expressing YFP-SspB_milli. As shown in Figure 3A, the YFP-SspB_milli band was at the position of approximately 100 kDa, indicating the dimeric status. We then introduced eight mutations (L6R, R9E, Y12Q, L13K, A16E, F17K, W20E, D23K), which has been reported to disturb hydrophobic interaction in the dimer-interface [30], into YFP-SspB_milli to generate the monomeric variant YFP-SspB_milli-8M. The YFP-SspB_milli-8M exhibited a monomeric state in native PAGE (Figure 3A). However, the YFP-SspB_milli-8M showed very low light-regulated binding ability to H1021-LOV-A in comparison to YFP-SspB_milli (Figure 3B), indicating that YFP-SspB_milli-8M cannot be used for optogenetic protein purification.

The eight mutations might not only affect the dimeric interface but also the monomeric conformation. We thus examined the SspB dimer structure to design new mutations. Lys^9^ could interact with Asp^100*^ (* indicates the residue in the dimer-related chain) through a hydrogen bond in the dimer interface (Supplementary Figure S1). Tyr^12^ formed a potential interaction with Asp^23*^ through a hydrogen bond in the dimer-interface and might also participate in maintaining the monomer structure by interactions with Arg^15^ and Asn^67^ (Supplementary Figure S1) [21,30]. Two Leu^13^ in antiparallel helices of the dimer interface formed hydrophobic interaction (Supplementary Figure S1). Accordingly, we designed two new mutants: YFP-SspB_nano-3AM (K9A, Y12A and L13A) and YFP-SspB_nano-3M (K9A, Y12L and L13V) (Figure 3C). The YFP-SspB_nano with high binding affinity was chosen as backbone, as new mutations might also decrease the binding abilities like the previous eight mutations. The mutant YFP-SspB_nano-3M was designed to preserve relatively long side chains, which might be important for structural integrity and functionality of the monomer.

We found that YFP-SspB_nano-3M showed slightly reduced binding ability in the light but much reduced binding in the dark compared with YFP-SspB_nano (Figure 3D). The YFP-SspB_nano-3AM showed much reduced binding ability in both the light and the dark conditions (Figure 3D), which met our hypothesis that mutation to small residues might influence the monomer structure and thus affect the binding ability to the SsrA part of H1021-LOV-A. More interestingly, the YFP-SspB_nano-3M/H1021-LOV-A pair showed more than two-fold enhanced purification efficacy in comparison with the previous YFP-SspB_milli/H1021-LOV-A pair (Figure 3E). The purified YFP-SspB_nano-3M and YFP-SspB_milli were then loaded in a native PAGE gel (Figure 3F). YFP-SspB_nano-3M exhibited a strong band at approximately 50 kDa and YFP-SspB_milli exhibited a strong band at approximately 100 kDa, indicating the monomer and dimer state, respectively. Similar mutations to the YFP-SspB_nano-3M were also introduced to YFP-SspB_micro and YFP-SspB_milli. However, both SspB_micro-3M and SspB_milli-3M showed much reduced binding abilities (Supplementary Figure S2). We then stuck to the newly engineered YFP-SspB_nano-3M/H1021-LOV-A pair for further studies and applications.

**Figure 4:**
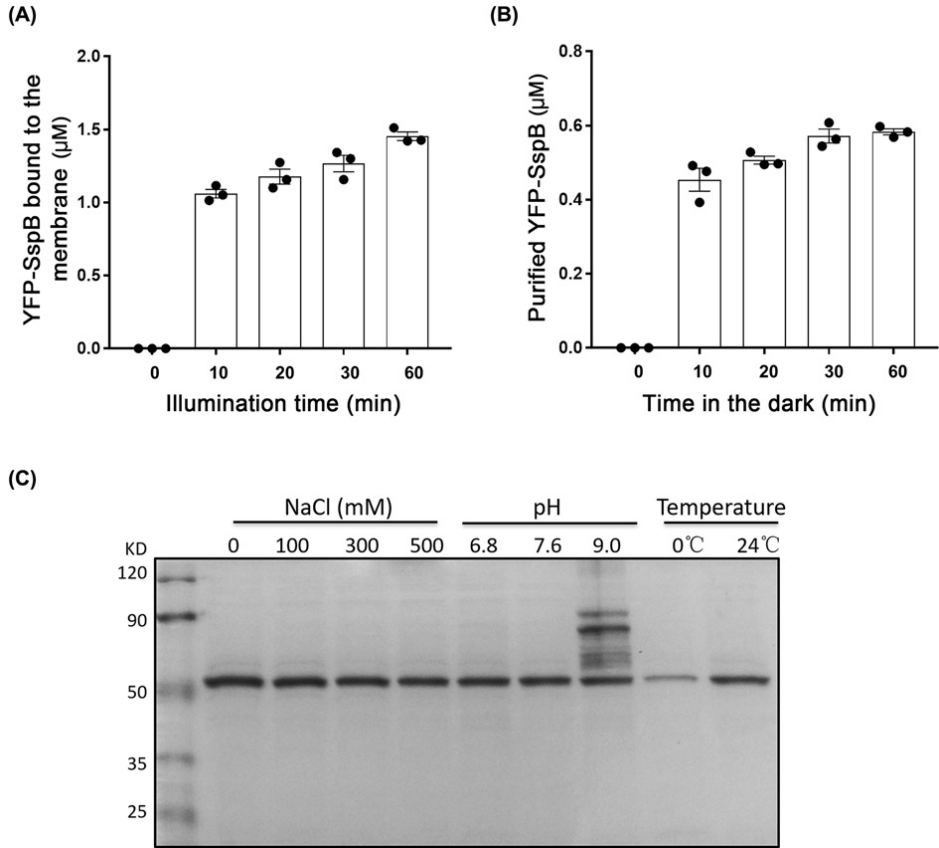
Characterizations of the H1021-LOV-A/SspB_nano-3M (**A**) Illumination time-dependent binding efficiency between H1021-LOV-A and YFP-SspB_nano-3M. Each reaction contained 4 μM YFP-SspB and membrane fraction from 5 ml *E. coli* in 300 μl buffer A. *n*=3, error bars = SEM, all individual data points are shown. (**B**) Time (in the dark)-dependent release efficiency of the YFP-SspB_nano-3M bound to H1021-LOV-A. The binding was stimulated by 20 min blue light. The released (purified) proteins were collected in 200 μl buffer A. *n*=3, error bars = SEM, all individual data points are shown. (**C**) Purification of YFP-SspB_nano-3M under different conditions. Each reaction contained 4 μM YFP-SspB and membrane fraction from 5 ml *E. coli* in 300 μl buffer A. Purified proteins were collected in 200 μl buffer A. A total of 20 μl samples were loaded in SDS/PAGE.

The required illumination and releasing time of YFP-SspB_nano-3M/H1021-LOV-A was then studied. We found that 10-min illumination for the binding and 10 min in the dark for the releasing were reasonable for the purification process (Figure 4A,B). Longer time only moderately increased the binding and releasing efficacy. Ten minutes for the binding and 10 min for the releasing can keep the purification time at approximately 40 min. A shorter preparation time might also help to keep the protein in a more native condition.

For the optogenetic purification method, only one buffer mimicking the physiological condition is needed throughout the whole process. The buffer conditions can also be modified accordingly. Salt concentration changes were well tolerated by this method (Figure 4C). It worked well at around physiological pH (6.8 and 7.6) but not at high pH 9.0 (Figure 4C). At 0°C, protein with high purity could still be obtained, albeit with reduced yield (approximately two-fold decreasing); arguing the applicability for purification of thermo-unstable proteins (Figure 4C).

### Applications of the optogenetic purification methods to other proteins

**Figure 5:**
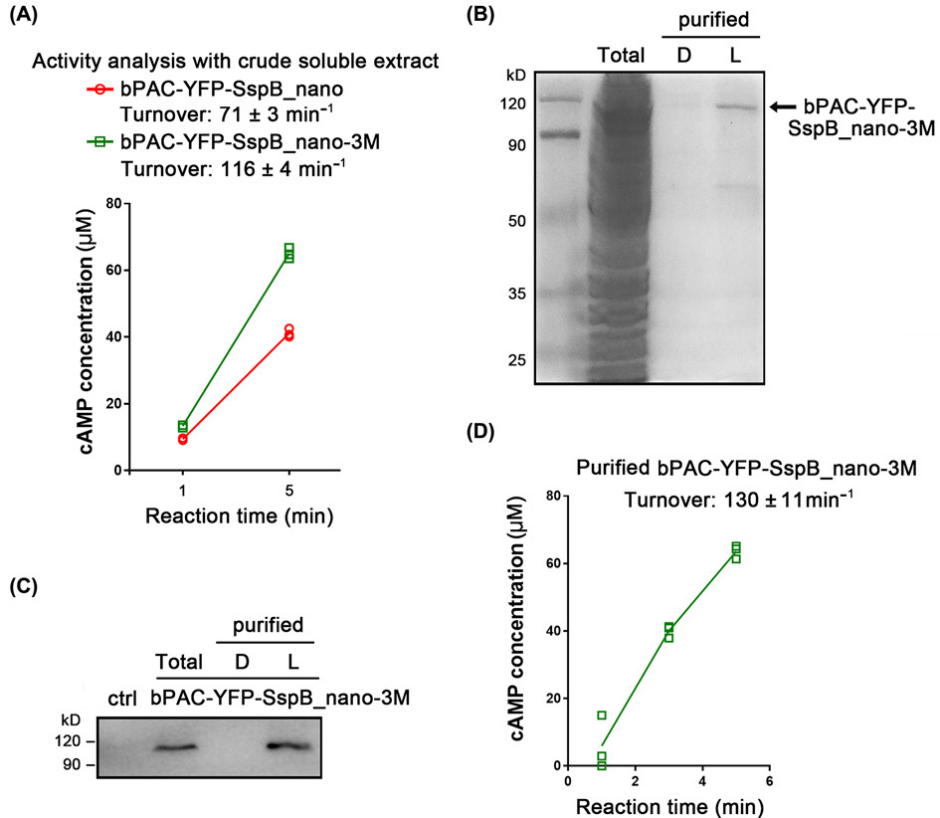
bPAC purification and activity analysis (**A**) Light-gated cAMP production abilities of bPAC-YFP-SspB_nano and bPAC-YFP-SspB_nano-3M. Reactions were illuminated by blue light (473 nm, 0.3 mW/mm^2^). Details of the *in vitro* reaction were described in the ‘Materials and methods’ part. *n*=3, error bars = SEM. All individual data points are shown. (**B**) The purified bPAC-YFP-SspB_nano-3M in SDS/PAGE. A total of 100 oocytes injected with bPAC-YFP-SspB_nano-3M were homogenized in 300 μl buffer, and then mixed with the membrane extract from 5 ml *E. coli* for the purification. The purified protein was collected in 200 μl buffer. Total: the total soluble extract from oocytes expressing bPAC-YFP-SspB_nano-3M, loading amount: 5 μl. D: released proteins after binding in the dark, loading amount: 20 μl. L: released proteins after binding in the light, loading amount: 20 μl. (**C**) Confirmation of the purified bPAC-YFP-SspB_nano-3M by Western blot. ctrl: oocyte without cRNA injection. Otherwise, the loading condition was similar to (C). Details of the Western blot were described in the ‘Materials and methods’ part. (**D**) cAMP production by purified bPAC-YFP-SspB_nano-3M. Samples were illuminated by blue light (473 nm, 0.3 mW/mm^2^). Details of the *in vitro* reaction were described in the ‘Materials and methods’ part. *n*=3, error bars = SEM. All individual data points are shown.

bPAC is a photo-activated adenylyl cyclase from the soil bacterium *Beggiatoa* and the most popular tool for optogenetic cAMP manipulation [28]. We fused YFP-SspB_nano and YFP-SspB_nano-3M to the C-terminal of bPAC and compared the activities of the fusion constructs. After 3 days’ expression in *Xenopus* oocyte, the total soluble fraction was extracted and used for *in vitro* reaction. The turnover of bPAC-YFP-SspB_nano in the light was determined to be ∼71 min^−1^ while for bPAC-YFP-SspB_nano-3M the turnover was ∼116 min^−1^ (Figure 5A), indicating that the dimerized version SspB_nano decreased the bPAC activity. We then purified bPAC-YFP-SspB_nano-3M after *Xenopus* oocyte expression using the H1021-LOV-A from *E. coli*. A dominant protein band of ∼100 kDa was observed in the SDS/PAGE (Figure 5B), and further confirmed to be bPAC-YFP-SspB_nano-3M by Western blot (Figure 5C). The molecular weight of bPAC-YFP-SspB_nano-3M was calculated to be ∼90 kDa, however its real size was more than 100 kDa in the SDS/PAGE, the bigger than calculated size of bPAC in the SDS/PAGE was also confirmed by previous studies [31]. The light activity of the purified bPAC-YFP-SspB_nano-3M was tested to be 130 min^−1^ (Figure 5D), which is close to the bPAC activity (156 min^−1^), detected by Lindner et al. [32] and SUMO-bPAC activity (108 min^−1^), detected by Stierl et al. [28].

**Figure 6:**
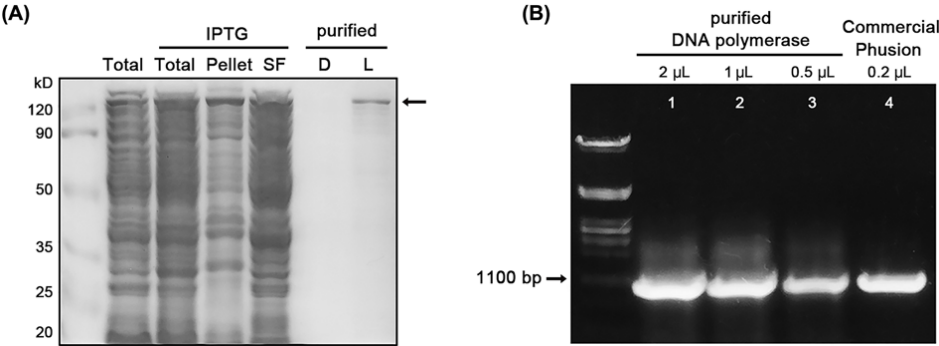
DNA polymerase purification and activity estimation (**A**) Confirmation of IPTG-induced SspB_nano-3M tagged DNA polymerase (∼120 kDa) expression and purification in SDS/PAGE. A total of 50 ml *E. coli* was homogenized by 1 ml buffer B, and the pellet part was resuspended by 1 ml buffer B before the Gel loading. Total: the whole cell lysate, loading amount: 5 μl. Pellet: sediment fraction, loading amount: 5 μl. SF: soluble fraction, loading amount: 5 μl. D: released proteins after binding in the dark, loading amount: 20 μl. L: released proteins after binding in the light, loading amount: 20 μl. (**B**) Agarose gel electrophoresis of PCR products with the purified and commercial DNA polymerase. Lanes 1–3: PCR products using 2, 1 and 0.5 μl of the purified DNA polymerase. Lane 4: PCR products using 0.2 μl (0.4 U) commercial Phusion DNA polymerase.

A DNA polymerase fused with SspB_nano-3M in its N-terminal was also expressed in *E. coli* BL21(DE3) for the purification. The extracted protein from 50 ml *E. coli* culture was diluted into 200 μl final volume. The purified polymerase-SspB_nano-3M was confirmed in the SDS/PAGE with the right size of ∼120 kDa (Figure 6A). Different amounts of purified DNA polymerase (2, 1 and 0.5 μl) were tested and compared with 0.2 μl (2 U/μl) commercial Thermo Fisher Scientific Phusion DNA polymerase (Figure 6B). The enzymatic activity of the purified DNA polymerase was estimated, based on the yield of the 1.1 kb PCR product. One microliter of purified DNA polymerase was estimated to be close to 0.4 U Thermo Fisher Scientific Phusion DNA polymerase, meaning that ∼80 units of functional DNA polymerase were produced from the 50 ml *E. coli* culture.

## Discussion

The iLID system is one of the most popular systems used for light-regulated protein interaction and widely used to regulate cell signaling and protein expression. In the present study, we chose the iLID system for optogenetic purification of soluble proteins because of several reasons: (1) well-studied and applied with several available mutants; (2) small size and (3) the chromophore of LOV domain protein, flavin mononucleotide (FMN), is ubiquitously available in many different cell types.

However, one potential problem is the strong homodimerization of the SspB part, which might interfere with the function of proteins fused to it. In the present study, we found that the bPAC activity is decreased when fused with the previously published dimeric SspB, possibly because the SspB dimer impaired the functionality of bPAC dimer (Figure 5A). McGinness et al. [30] first introduced eight mutations in the N-terminal α helix of SspB (SspB_nano-8M in the present study) to abolish the dimerization. The binding ability of SspB_nano-8M with SsrA became 3-fold weaker than wild type SspB due to the slightly hydrophobic groove distortion. However, we found that the binding affinity between SspB_nano-8M and LOV-A was too low to be used for protein precipitation (Figure 3B). Moreover, truncation of the N-terminal α helix (25 aa) of SspB totally abolished its binding to SsrA, suggesting that the α helix of SspB not only participates in dimer formation, but also contributes to the conformational stability of SspB monomer. We then designed a new mutant by introducing only three mutations to SspB (SspB_nano-3M in the present study). The SspB_nano-3M exhibited a monomeric state in native-PAGE (Figure 3F). Compared with the previous SspB, SspB_nano-3M showed no influence on the protein activity when fused with bPAC (Figure 5). In addition, the SspB_nano-3M displayed an increased light regulation efficacy (Figure 3E), which makes it ideal not only for protein purification but also for future optogenetic applications.

Recently, Horner et al. have used the PhyB/PIF system to isolate the tyrosine kinase ZAP70 [33]. Biotinylated PhyB is immobilized on NeutrAvidin (N)-functionalized agarose beads to pull down the ZAP70-PIF fusion protein when illuminated with 660 nm red light and released when exposed to 740 nm far-red light. The PhyB requires phycocyanobilin as chromophore, which is not naturally present in many organisms, including mammals [34]. We chose the iLID system in this study partially because of its ubiquitously available chromophore, FMN. Furthermore, we make use of the biological membranes instead of columns or beads. We have applied two ideal membrane anchors for *Xenopus* oocyte and *E. coli*. The membrane fractions can be easily separated by routine centrifugation. *E. coli* contains the inner membrane and outer membrane, and both tend to form stable vesicles after disruption by sonication, which are native platforms for protein purifications [35]. With all materials from the biological system, the optogenetic protein purification method mem-iLID can work under physiological conditions and does not require buffer changing for protein elution, which can sometime damage the POI or cause non-specificity. The purification efficiency with the mem-iLID is however lower than the traditional method, 10–15% of the total input protein can be obtained from our optogenentic method. In the present study, we used a relatively small and flexible scale. Large-scale production of proteins can be achieved by increasing the input amount.

In sum, the newly developed mem-iLID method is easy, fast, economic, flexible in scale and can work under physiological conditions. Furthermore, the mem-iLID can tolerate salt concentration changes (Figure 4C). The purification process can also be operated on ice at 0°C when necessary (Figure 4C). This easy and economic method will facilitate extensive protein engineering and functional studies. The newly designed SspB variant could also improve other iLID-based optogenetic applications.

## Materials and methods

### DNA and plasmids

The iLID [13] consisting of LOV-A (*As*LOV2-SsrA) and SspB and the DNA polymerase (*Pfu*-Sso7d) gene were synthesized by GeneArt Strings DNA fragments (Life Technologies, Thermo Fisher Scientific, Darmstadt) according to the published DNA sequences. The Lyn11 sequence and H1021 sequence were ordered as DNA primers and fused to iLID by PCR. The bPAC, YFP, pGEMHE vector and pET28b vector were from the lab stock. The designed constructs in Figure 1A were inserted into the *Xenopus* oocyte expression vector pGEMHE within N-terminal BamHI and C-terminal HandIII restriction sites. The constructs used for *E. coli* expression were cloned into pET28b vector within N-terminal NcoI and C-terminal HandIII restriction sites. Mutations of SspB_micro and SspB_milli were made by QuikChange Site-Directed Mutagenesis. All DNA sequences were confirmed by sequencing.

### RNA generation for *Xenopus* oocyte expression

The cloned pGEMHE plasmids containing different constructs were linearized by NheI digestion and used for the *in vitro* generation of cRNAs with the AmpliCap-MaxT7 High Yield Message Maker Kit (Epicentre Biotechnologies, Madison). Thirty nanogram of cRNA (otherwise indicated in the figure) of different constructs were injected to *Xenopus* oocytes by Nanoject III (Drummond Scientific Company, Broomall). The oocytes were then incubated in ND96 buffer (96 mM NaCl, 2 mM KCl, 1 mM CaCl_2_, 1 mM MgCl_2_, 5 mM HEPES, pH 7.6) at 17°C for 3 days before use.

### Extractions of membrane and soluble fractions from *Xenopus* oocytes

Fifty *Xenopus* oocytes expressing soluble protein were pooled and homogenized in 1.5 ml Eppendorf tube with 300 μl buffer A (75 mM Tris-HCl, 5 mM MgCl_2_ and 100 mM NaCl, pH 7.6) simply by pipetting using an Eppendorf pipette with a 10–100 μl tip. The homogenate was centrifuged at 15000×***g*** for 10 min at 4°C. The supernatant was collected and transferred to a fresh tube as the soluble fraction for future use.

Membrane fractions of oocytes expressing Lyn11-associated proteins were isolated by two-step centrifugation. After the homogenization step mentioned before, we first used 500×***g*** centrifugation (5 min) to remove the big pellets, yolk and cell debris. The supernatant was then transferred to a fresh tube and centrifuged at 15000×***g*** for 1.5 min. The sediment as the membrane fraction was harvested and washed three times by 300 μl buffer A. At last, membrane extracts from 25 oocytes were kept as pellet in a 1.5-ml Eppendorf tube after centrifugation (15000×***g***, 1.5 min) for future use.

### Protein expression and membrane/soluble extraction from *E. coli*

Different pET28b-YFP-SspB plasmids and pET28b-H1021-LOV-A were transformed into *E. coli* BL21(DE3). Positive transformants were picked and pre-cultured in 5 ml LB medium with 50 μg/ml kanamycin overnight at 37°C. Then 1 ml LB medium was inoculated into 100 ml fresh LB medium and cultured for ∼4 h at 37°C to reach an OD_600_ value of ∼0.6. A total of 0.5 mM IPTG was added into LB medium to induce protein expression and the temperature was changed to 28°C for overnight culture.

The cells from 50 ml medium were then harvested by centrifugation at 2000×***g*** for 5 min, washed three times and resuspended in 1 ml buffer A. The cells were disrupted by an ultrasonic processor UP50H at 100% power (working 0.4 s per cycle) for 20 min on ice. To collect the soluble fractions, the lysed mixture was centrifuged at 15000×***g***, 4°C for 30 min, and the supernatant was transferred to a fresh tube as the soluble fraction. The lysed mixture containing membrane proteins H1021-LOV-A was first centrifuged at 500**×*g*** for 2 min and the supernatant was transferred to a fresh tube. After a second centrifugation at 15000×***g*** for 1 min, the supernatant was discarded and the membrane fraction containing H1021-LOV-A was resuspended and washed three times. Membrane extracts from 50 ml *E. coli* culture were resuspended with 200 μl buffer A. The amount of membrane extract used for protein purification was indicated in the figure.

### Light-assisted purification of proteins

For purifications with *Xenopus* oocyte extracts, the contents of target proteins in the soluble extract were quantified by fluorescence emission, and then 300 μl soluble extract (after dilution to desired target protein concentration) was used to resuspend the extracted membrane pellet (from 25 *Xenopus* oocytes) in a 1.5-ml Eppendorf tube (Figure 1A). The mixture was stimulated by 10, 20 or 30 min (see different figures) blue light (470 nm LED at 100 μW/mm^2^, otherwise indicated in the figure) to induce the binding. After binding, the mixture was centrifuged at 15000×***g*** for 1 min and washed three times by 300 μl buffer A, 1 min 15000×***g*** centrifugation was applied after each wash step. The final membrane pellet was resuspended by 300 μl fresh buffer A and placed in the dark for 10, 20 or 30 min (see different figures) to release the bound target protein. The released (purified) protein in the supernatant was collected by 15000×***g*** centrifugation at 4°C for 20 min (5 min was used for fast preparations), and transferred into a fresh tube for further test.

The purification process with the *E. coli* system is very similar to above. Only the amounts of soluble extract and membrane extract were changed (for details, see the figure legends part).

For bPAC purification, 100 injected *Xenopus* oocytes were homogenized in 300 μl buffer A containing 5 mM DTT and 1× Protease Inhibitor Cocktail (Roche, Basel), which could stabilize bPAC protein. The soluble extract was collected as mentioned before, and used to resuspend the membrane extract from 5 ml *E. coli*. After purification, the purified bPAC was collected in 200 μl buffer A containing 5 mM DTT and 1× Protease Inhibitor Cocktail (Roche, Basel).

For the DNA polymerase purification, buffer B (75 mM Tris-HCl, pH 7.6, 5 mM MgCl_2_ and 300 mM KCl) was used to increase its solubility. To store the purified SspB_nano-3M-tagged DNA polymerase, the buffer was then dialyzed using an ultra-centrifugal filter tube (Amicon® Ultra-0.5 50k device, Millipore, Darmstadt) extensively with buffer C (100 mM Tris-HCl pH 8.0, 0.2 mM EDTA, 0.2% NP-40, 0.2% Tween 20, and 2 mM DTT). During purification, the membrane extract from 5 ml *E. coli* was resuspended by 300 μl soluble extract without dilution. At last, the total amount of the purified DNA polymerase from 50 ml *E. coli* culture was collected in 200 μl storage buffer (mixture of 100 μl buffer C + 100 μl glycerol).

### Protein quantification by fluorescence

The YFP-tagged proteins were quantified by the relative fluorescence units (RFUs). Purified YFP protein standard (10 μg, RayBiotech, Norcross) was diluted to 2500 ng/ml in buffer A to calibrate the fluorometer. Fluorescence emission was measured at the range of 510–580 nm using the Quantus™ Fluorometer (Promega, Walldorf) with 495-nm excitation.

### Native PAGE, SDS/PAGE and Western blot

The whole lysates, crude extracts or purified proteins were checked in 10% native PAGE or SDS/PAGE for dimeric state and purity, respectively. The gel was stained by EZBlue (Sigma, Darmstadt) for 30 min and washed by water according to its protocol.

For Western blot, the protein samples were loaded on to a 10% SDS/PAGE for electrophoresis and transferred into PVDF membranes (Millipore, Darmstadt). Membranes were blocked in Tris-buffered saline with Tween 20 (TBST: 1 mM Tris-HCl, 150 mM NaCl, 0.05% Tween-20, pH 7.4) containing 5% BSA for 0.5 h and subsequently incubated overnight at 4°C with diluted primary antibody against YFP with HRP conjugation (1:2000 dilution, Life Technologies, Thermo Fisher Scientific, Darmstadt). The band was imaged using the Odyssey Fc Imaging System from LI-COR Biosciences.

### *In vitro* reaction of bPAC activity and cAMP detection

For the *in vitro* reaction to test the bPAC activity, 10 injected oocytes were homogenized by 100 μl buffer A containing 5 mM DTT and 1× Protease Inhibitor Cocktail (Roche, Basel). The soluble extract was collected as mentioned before, and then dialyzed by using an ultra-centrifugal filter tube (Amicon® Ultra-0.5 30k device, Millipore, Darmstadt) to remove the accumulated cAMP/cGMP from the *Xenopus* oocyte cytosol. To start the reaction, 0.1 μl of 100 mM ATP stock was added into 10 μl soluble extract or purified bPAC to reach the final concentration of 1 mM. The bPAC light activity was stimulated by a blue light laser (473 nm, 0.3 mW/mm^2^). A total of 190 μl sample diluent (containing 0.1 M HCl) was used to stop the 10 μl reaction mix and stabilize the produced cAMP. The cAMP assay was performed with DetectX High Sensitivity Direct Cyclic cAMP Chemiluminescent Immunoassay Kit (Arbor Assays, Ann Arbor).

### Functional assay of DNA polymerase based on PCR amplification

The YFP-SspB DNA with the size of ∼1100 bp was used as the template to assess the activity of the purified DNA polymerase. Different amounts of purified DNA polymerase (2, 1 and 0.5 μl) were added to 20 μl PCR reactions separately. The commercial Phusion High-Fidelity DNA Polymerase (Thermo Fisher Scientific, Darmstadt) was used to estimate the enzymatic activity of purified DNA polymerase. Other components of the PCR reactions like dNTPs and buffers were added according to the protocol of the Phusion High-Fidelity DNA Polymerase.

### Data analysis

Results are presented as mean ± standard error of the mean (SEM) with GraphPad Prism software (San Diego, U.S.A.).

## Supplementary Material

Supplementary Figures S1-S2

## Data Availability

All relevant source data are provided as additional files. All DNA and plasmids are available from the corresponding authors on reasonable requests.

## Abbreviations

bPAC: bacteria photo-activated adenylyl cyclase
Cry2: cryptochrome 2
FMN: flavin mononucleotide
iLID: improved light-induced dimer
IPTG: isopropyl-β-d-thiogalactopyranoside
LOV: light, oxygen, or voltage
PhyB: phytochrome B
POI: protein of interest
