# Supplementary material for: mem-iLID, a fast and economic protein purification method
Source: Biosci Rep. 2021 Jun 28;41(7):BSR20210800. doi: 10.1042/BSR20210800 (PMC8239496; doi:10.1042/BSR20210800)
Supplement: Supplementary Figures S1-S2 [file BSR-2021-0800_supp.pdf]

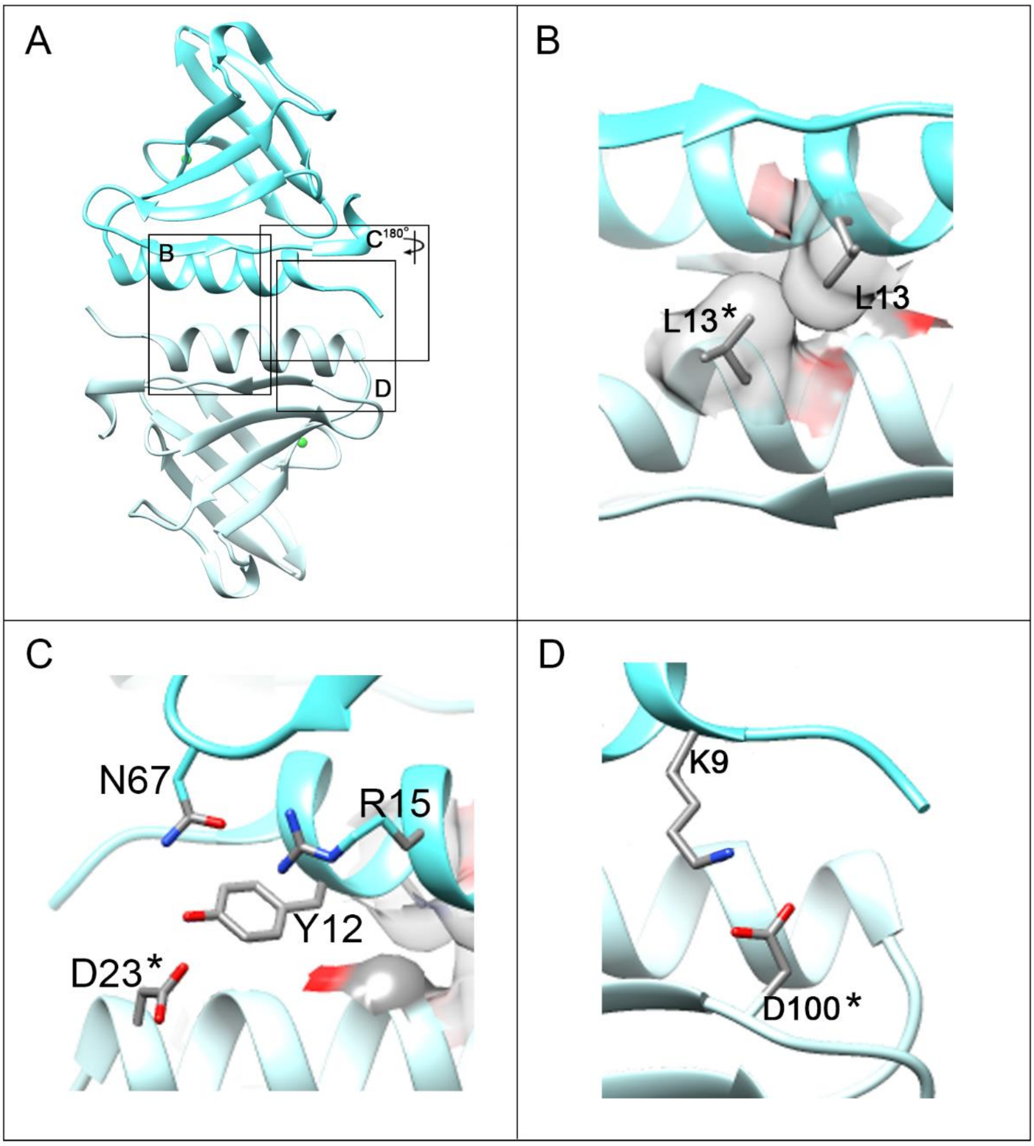

Figure S1. Structure of dimeric SspB yields insight to the mutated residues.

(A) The dimeric SspB complex in antiparallel form. (generated from PDB ID: 1ZSZ) **(B)** The hydrophobic interaction between Leu13 and Leu13* in the dimer-interface. **(C)** The Tyr12 form a potential interaction with Asp23* through a hydrogen bond in the dimer-interface and might also participate in maintaining the monomer structure by interactions with Asn67 and Arg15. **(D)** The Lys9 interacted with Asp100* through a hydrogen bond in the dimer-interface. (*: amino acid from the antiparallel SspB)

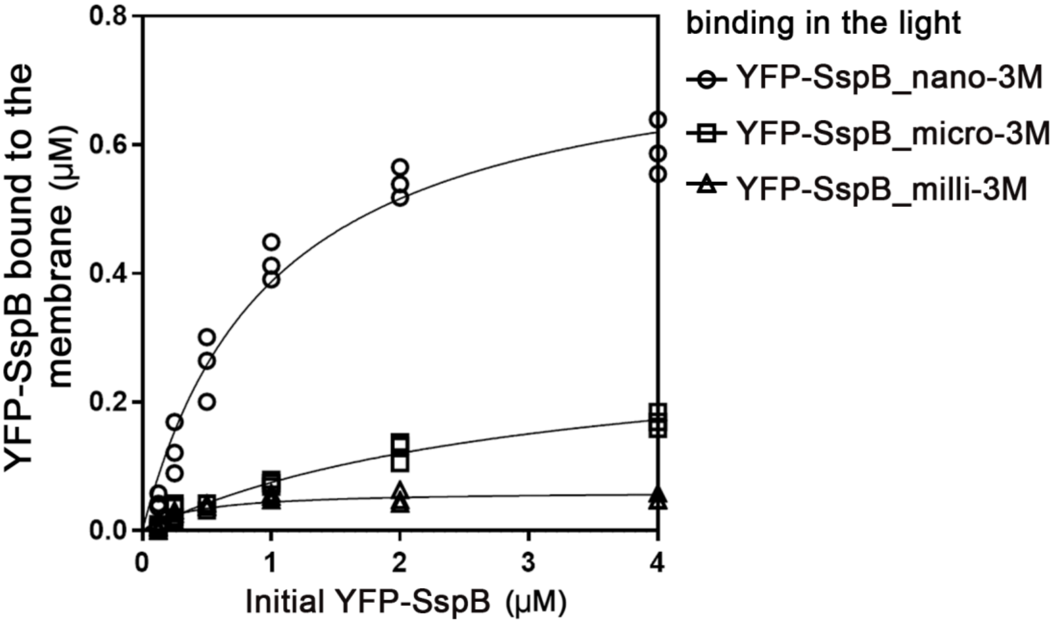

Figure S2. The binding efficacy comparison of different monomeric SspB mutants. Comparing the binding abilities of different YFP-SspB mutants to H1021-LOV-A after 20 min blue light illumination. In each reaction, the membrane fraction from 2.5 ml *E. coli* was mixed with different concentrations of YFP-SspB mutants in 300 μl buffer A. Michaelis-Menten curves were fitted. n = 3, error bars = SEM. All individual data points are shown.
